# Salicylic acid and jasmonic acid in plant immunity

**DOI:** 10.1093/hr/uhaf082

**Published:** 2025-03-11

**Authors:** Pingyu Zhang, Edan Jackson, Xin Li, Yuelin Zhang

**Affiliations:** College of Life Sciences, Sichuan University, Chengdu 610064, China; Michael Smith Laboratories, University of British Columbia, Vancouver, BC V6T 1Z4, Canada; Department of Botany, University of British Columbia, Vancouver, BC V6T 1Z4, Canada; Michael Smith Laboratories, University of British Columbia, Vancouver, BC V6T 1Z4, Canada; Department of Botany, University of British Columbia, Vancouver, BC V6T 1Z4, Canada; College of Life Sciences, Sichuan University, Chengdu 610064, China

## Abstract

Salicylic acid (SA) and jasmonic acid (JA) are the two most important phytohormones in plant immunity. While SA plays pivotal roles in local and systemic acquired resistance (SAR) against biotrophic pathogens, JA, on the other hand, contributes to defense against necrotrophic pathogens, herbivores, and induced systemic resistance (ISR). Over the past 30 years, extensive research has elucidated the biosynthesis, metabolism, physiological functions, and signaling of both SA and JA. Here, we present an overview of signaling pathways of SA and JA and how they interact with each other to fine-tune plant defense responses.

## Introduction

Land plants have evolved an intricate defense system to cope with frequent pathogen infections, with two major layers of plant immunity extensively studied. The first layer is mediated by cell surface-localized pattern recognition receptors (PRRs), which recognize conserved pathogen-associated molecular patterns (PAMPs), turning on pattern-triggered immunity (PTI) [1]. However, to successfully infect plants, pathogens have evolved to weaken or evade PTI by delivering effector molecules to activate effector-triggered susceptibility. To counter such virulence strategy, plants have evolved a second layer of defense involving intracellular immune receptors, primarily nucleotide-binding leucine-rich repeat receptors (NLRs), which directly or indirectly sense the presence of effectors, thereby activating effector-triggered immunity (ETI) [2]. ETI is a manifestation of gene-for-gene resistance, where a single-plant resistance (*R*) gene confers immunity to pathogens carrying the corresponding avirulence (*Avr*) gene. ETI usually involves programmed cell death at the site of infection, known as the hypersensitive response (HR), which effectively blocks the intrusion of biotrophic pathogens relying on living host tissues for survival [3]. Moreover, the local activation of PTI and ETI responses can trigger enhanced resistance in uninfected parts of the plant, a phenomenon known as SAR, which confers long-lasting and broad-spectrum immunity against pathogens [3, 4].

Local defense often promotes the biosynthesis of a series of phytohormones, thus activating their signaling pathways. Among them, salicylic acid (SA) and jasmonic acid (JA) are the two most crucial signals for plant immunity. Plants rely on SA to ward off biotrophic and hemibiotrophic pathogens, whereas JA-induced responses primarily contribute to defense against necrotrophic pathogens and herbivores, as well as wounding. For a long time, it was commonly accepted that SA and JA generally act antagonistically. However, emerging evidence now indicates that their crosstalk can also be synergistic [5–9]. The intricate interplay between SA and JA equips plants with a resilient and adaptable immune system, but it can also be exploited by pathogens to attenuate host defenses. Here, we focus on reviewing the biosynthesis and metabolism of SA and JA, their signaling pathways, and the crosstalk between them in plant immunity.

## SA biosynthesis and metabolism

**Figure 1:**
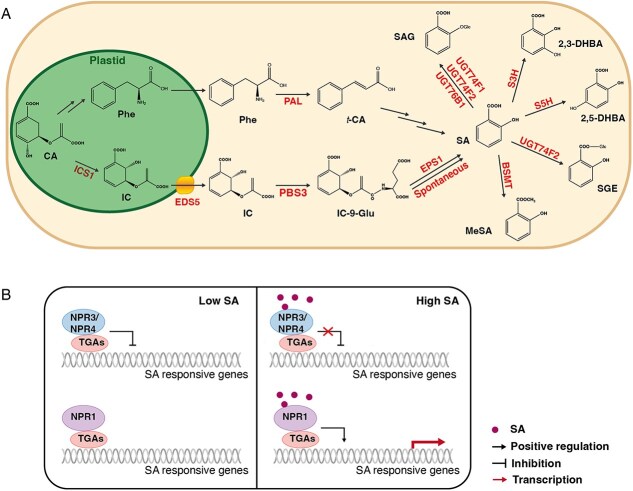
The biosynthesis, metabolism and signaling of SA. (A) Both isochorismate synthase (ICS) and phenylalanine ammonia lyase (PAL) pathways start from chorismate. For the proposed PAL pathway, SA can be synthesized from Phe through a series of enzymatic reactions. In *Arabidopsis*, most pathogen-induced SA is generated from ICS pathway. Chorismate (CA) is converted to isochorismate (IC) by ICS1/ICS2. IC is then transported to cytosol by the MATE transporter ENHANCED DISEASE SUSCEPTIBILITY 5 (EDS5), where IC is converted to SA. To regulate SA levels, SA undergoes different chemical modifications, including glycosylation, methylation and hydroxylation. (B) When SA levels are low in the uninfected state, NPR3/4 interact with TGA2/5/6 to inhibit the expression of SA-induced defense genes. While in the presence of elevated SA levels, the transcriptional repression activities of NPR3/4 have been suppressed. At the same time, binding of SA promotes the transcriptional activation of NPR1, which recruits transcription factors to induce expression of defense-related genes. PBS3, AVRPPHB SUSCEPTIBLE 3 (PBS3); IC-9-Glu, isochorismate-9-glutamate; EPS1, ENHANCED *PSEUDOMONAS* SUSCEPTIBILITY 1; BSMT1, SA METHYL TRANSFERASE 1; PAL, PHENYLALANINE AMMONIA-LYASE; S3H, SALICYLIC ACID 3-HYDROXYLASE; S5H, SALICYLIC ACID 5-HYDROXYLASE.

It is widely accepted that plants possess two independent pathways to synthesize SA: the isochorismate synthase (ICS) and phenylalanine ammonia lyase (PAL) pathways (Fig. 1A) [10, 11]. Both pathways utilize chloroplast-produced chorismite as precursors, but different plant species employ these pathways to different degrees. For instance, the ICS pathway is the primary contributor for pathogen-induced SA in *Arabidopsis thaliana* (hereafter, *Arabidopsis*) [12, 13]. By contrast, in *Glycine max*, ICS and PAL pathways are equally used for defense-related SA biosynthesis [14]. In tobacco, however, the expression levels of *PAL*, but not *ICS*, and the PAL enzymatic activity increased drastically during TMV infection, implying that the PAL pathway is the primary one contributing to tobacco SA production [15].

In addition to ICS, two other proteins, ENHANCED DISEASE SUSCEPTIBILITY 5 (EDS5) and AVRPPHB SUSCEPTIBLE 3 (PBS3), also contribute to SA production in *Arabidopsis* (Fig. 1A) [16–20]*.* EDS5 belongs to the multidrug and toxin extrusion (MATE) transporter family. It localizes on the chloroplast envelope [21] and likely transports isochorismate (IC) from plastid to cytosol [22, 23]. PBS3 is a member of the GRETCHEN HAGEN 3 (GH3) family of acyl-adenylate/thioester-forming enzymes, which can conjugate phytohormone acyl substrates to amino acids *in vitro* [17, 18]. PBS3 catalyzes the conjugation of l-glutamate to IC, yielding the key intermediate isochorismate-9-glutamate (IC-9-Glu) [17, 18]. This intermediate then spontaneously decomposes to SA [22]. Moreover, another study reported that ENHANCED PSEUDOMONAS SUSCEPTIBILITY 1 (EPS1) can stimulate the conversion of IC-9-Glu into SA [23]. Interestingly, phylogenetic analysis revealed that EPS1 belongs to a unique clade of BAHD acyltransferases only presented in the Brassicaceae family, indicating that it is a newly recruited to the SA biosynthetic pathway [23]. With the completion of the ICS pathway, it is clear that pathogen infection strongly induces SA biosynthetic genes, and such induction is coordinately regulated by the transcription factors SAR DEFICIENT 1 (SARD1) and CALMODULIN BINDING PROTEIN 60-LIKE G (CBP60g), which are essential for pathogen-induced SA biosynthesis in *Arabidopsis* [24, 25].

Although the ICS pathway for SA production in plants is a relatively recent discovery, the PAL pathway has been recognized much earlier [26]. PAL is the entrance enzyme in the PAL pathway that converts phenylalanine (Phe) to *trans*-cinnamic acid (*t*-CA) (Fig. 1A) [27]. Labeling studies in tobacco indicated that SA can be formed from *t*-CA via benzoic acid (BA) [26]. Multiple lines of evidence suggest that PALs contribute to SA biosynthesis in plants. In the quadruple mutants of all four Arabidopsis *PAL* genes, the basal and the pathogen-induced SA levels were reduced to 25% and 50% of that in wild type, respectively [28]. In soybean, silencing either *PAL* or *ICS* was sufficient to suppress the SA accumulation [14]. Meanwhile, the SA content of rice *OsPAL6* knockout mutant was significantly decreased compared to the wild type [29]. By analyzing mutants of Arabidopsis, an additional crucial component of the PAL pathway, ABNORMAL INFLORESCENCE MERISTEM1 (AIM1) was identified and later shown to be important for rice SA biosynthesis [30]. AIM1-dependent β-oxidation enzymes function in conversion of *t*-CA into BA [30, 31]. About 30 years ago, it was proposed that the last step in converting BA into SA is catalyzed by a hypothetical BENZOIC ACID 2-HYDROXYLASE (BA2H) [32]. However, this enzyme has not been identified yet, perhaps due to a very wide range of enzymes that could potentially fulfill this role. Intriguingly, a recent isotopic labeling study to investigate the role of the PAL pathway in SA biosynthesis suggested that this pathway contributes to the synthesis of 4-HBA rather than SA in *Arabidopsis*, indicating that SA could be converted from BA generated independently of the PALs [33].

Once synthesized, SA can undergo various modifications, including hydroxylation, glycosylation, methylation, and amino acid conjugation, which typically deactivate SA and help fine-tune its homeostasis (Fig. 1A) [10, 34–36]. There are two forms of SA glucosides, SA 2-*O*-β-d-glucoside (SAG) and SA glucose ester (SGE). In *Arabidopsis*, UGT74F1, UGT74F2, and UGT76B1 facilitate the transfer of a glucosyl group from UDP-glucose to the hydroxyl group of SA to produce SAG [37–40]. In addition, UGT74F2 can transfer glucose to the carboxyl group of SA to produce SGE [37–40]. Recently, CsUGT87E7 was reported to glycosylate SA to form SGE and play a positive role in plant disease resistance in *Camellia sinensis* [41]. In Arabidopsis *ugt74f2* and *ugt76b1* mutants, an enhanced disease resistance phenotype was observed, suggesting that UGT74F2 and UGT76B1 play negative roles in plant immunity [39, 40, 42–44]. Furthermore, SA can be converted to the gaseous methyl-SA (MeSA) by an SA methyl transferase after herbivory attacks [45]. MeSA is an important constitute of floral scents and has been proposed as an airborne signal involved in plant-to-plant communication [46]. Recently, it was shown that MeSA can be converted to SA by SALICYLIC ACID-BINDING PROTEIN-2 (SABP2) in neighboring plants to activate defense responses in tobacco [47]. SA can also be inactivated by SA 3-HYDROXYLASE (S3H/DLO1) and SA 5-HYDROXYLASE (S5H/DMR6) to produce 2,3-DHBA and 2,5-DHBA, respectively [48, 49]. Accordingly, mutations in these hydroxylases resulted in increased SA levels and enhanced pathogen resistance [48, 49].

## SA signaling

As a key plant hormone that mediates host responses against pathogens, SA strongly induces the expression of defense marker *pathogenesis-related* (*PR*) genes [50–52]. To identify components for SA perception, several independent forward genetic screens were carried out [53–55]. Notably, NONEXPRESSOR OF PATHOGENESIS-RELATED GENES 1 (NPR1), a central regulator of SA-mediated defense, was identified by screening mutants defective in SA-induced expression of *PR* genes or pathogen resistance [53–55]. Overexpression of *NPR1* in diverse plant species increases resistance to pathogens, indicating its crucial role in connecting SA and downstream defense reponses [56–62]. NPR1 contains an N-terminal broad complex, Tramtrack and Bric-à-brac and zinc finger (BTB/POZ) region, four ankyrin repeats in the middle, and an SA-binding domain at the C-terminus [63–65]. Extensive evidence suggests that NPR1 serves as a transcription coactivator by interacting with TGACG SEQUENCE-SPECIFIC BINDING PROTEIN (TGA) and associating with histone acetyltransferases (HAC) (Fig. 1B) [66–69]. Knockout analysis of TGA transcription factor genes revealed that TGA2, TGA5, and TGA6 function redundantly in the SA-induced *PR* gene expression and pathogen resistance [68]. Structural analysis revealed that NPR1 forms a bird-shaped homodimer, with the BTBs in the middle and the ANKs forming the wings, and activates downstream gene expression by bridging two TGA complexes [64].

Two paralogs of NPR1, NPR3 and NPR4, act as bona fide SA receptors [64, 70, 71]. Knockout analysis showed that NRP3 and NPR4 have partially redundant functions in negative regulation of immunity in *Arabidopsis* [72]. In the absence of SA, NRP3/NPR4 work together with TGA2, TGA5, and TGA6 to suppress the expression of *PR* genes (Fig. 1B) [68]. NRP3/NPR4 were initially proposed as CUL3 adaptors mediating NPR1 degradation [70]. However, epistasis analysis argued against this hypothesis, since NRP3/NPR4 function in parallel with NPR1 [71]. In this regard, Ding et al. demonstrated that NPR1 and NRP3/NPR4 play opposite roles in the transcriptional regulation of plant immunity, with NRP3/NPR4 function as transcriptional repressors [71]. An EAR motif at C-terminus of NRP3/NPR4 is required for their transcriptional repression activity, and mutation of this motif in NPR4 eliminates its ability to repress *SARD1* and *WRKY70* [71]. In addition, *npr4-4D*, a gain-of-function mutant with a single amino acid change that abolishes the SA-binding ability of NPR4, exhibits similar SA-insensitivity phenotypes as *npr1* mutants [71]. Furthermore, double mutant analysis revealed that *npr1* and *npr4-4D* mutants have additive effects on PTI, ETI, and SA-induced gene expression [73]. Therefore, the current model for SA signaling is that NPR1 and NPR3/NPR4 have opposite roles in the transcription regulation of plant defense against pathogens. As SA levels rise during pathogen infection, its binding to NPR3/NPR4 releases transcriptional repression of defense genes during pathogen infection, whereas the binding of SA to NPR1 further activates the expression of defense genes [71].

## SA in plant immunity

Early evidence supporting SA's role in defense came from analysis of plants overexpressing the bacterial salicylate hydroxylase gene *NahG*, which catalyzes SA degradation [74]. *NahG* transgenic Arabidopsis plants failed to accumulate SA and showed increased susceptibility to both virulent *Pseudomonas syringae* pv*. tomato* (*Pst*) DC3000 and the avirulent strain *Pst* DC3000 *AvrRpt2* [74–76]. In *NahG* transgenic tobacco, *N* gene-mediated ETI against TMV is severely compromised [74]. Application of the synthetic SA analog INA restored disease resistance in *NahG* transgenic plants [74]. Studies of the SA-deficient Arabidopsis *sid2* (an *ICS1* loss-of-function allele) and *eds5* mutants further confirmed the importance of SA in plant basal resistance as they displayed lower SA levels, decreased *PR* genes expression, and enhanced susceptibility after pathogen infection [13, 20]. Similarly, blocking SA accumulation by overexpression of enzymes involved in SA metabolism, such as *S3H*, *S5H,* and *BSMT1* also leads to enhanced susceptibility to pathogens [45, 48, 49, 77].

Unlike Arabidopsis, rice has constitutively high SA levels. Although no obvious SA increase was observed after inoculation of the fungal pathogen *Magnaporthe grisea* [78], many studies also demonstrated that SA is a key defense hormone in rice. For instance, *NahG*-expressing rice exhibited increased susceptibility to *M. grisea* [79]. In addition, overexpression of *AtNPR1* or its rice homolog in rice boosts resistance to the bacterial blight-causing *Xanthomonas oryzae* pv. *oryzae* (*Xoo*), suggesting that components of SA signal transduction, rather than the SA accumulation, are the limiting factors of SA responses in rice [80, 81]*.* Silencing *GmPAL* or *GmICS* leads to decreased SA levels and compromised basal resistance and ETI against *P. syringae* pv*. glycinea* (*Psg*) in soybean [14]. In wheat, infection of *Fusarium graminearum* also triggers a considerable accumulation of SA and the expression SA-related defense genes [82]. To counteract the SA-mediated immunity, *F. graminearum* encodes a NahG, which decreases the endogenous SA content of wheat during infection, thereby weakening SA-mediated immunity [83]. Mutation of *FgNahG* lead to enhanced sensitivity to SA and increased accumulation of SA [83]. Taken together, these findings indicate that SA is critical for disease resistance in a wide range of plants.

SA also plays a critical role in both PTI and ETI [10, 11, 73, 84, 85]. SA treatment enhances the expression of PAMP receptors and other PTI components, such as FLAGELLIN SENSITIVE 2 (FLS2), ETHYLENE RESPONSE FACTOR (ERF), CHITIN ELICITOR RECEPTOR KINASE 1 (CERK1), SUPPRESSOR OF BIR 1-1 (SOBIR1), MITOGEN-ACTIVATED PROTEIN KINASES (MAPKs), calcium-dependent kinases, and RESPIRATORY BURST OXIDASE HOMOLOG PROTEIN D (RbohD) [11, 84, 86–89]. Furthermore, SA can induce the expression the master transcription factors SARD1 and CBP60g, which directly bind and activate the expression of the positive regulators of PTI, including BRI1-ASSOCIATED RECEPTOR KINASE 1 (BAK1), BAK1-LIKE 1 (BKK1), BOTRYTIS-INDUCED KINASE 1 (BIK1), and MITOGEN-ACTIVATED PROTEIN KINASE 4 (MKK4), etc. [25]. Blocking the biosynthesis or perception of SA results in enhanced susceptibility to *Pst* DC3000 *hrcC^−^* and *Pst* DC3000 [71, 73, 85], as growth of these bacteria on *npr1-1 npr4-4D* mutants is significantly higher than on WT, indicating that NPR1/NPR3/NPR4-dependent SA signaling is required for boosting PTI [71, 73, 85].

Similarly, amplification of ETI also relies on the upregulation of SA biosynthesis and activation of SA signaling [70, 90, 91]. Arabidopsis plants expressing *NahG* and *sid2* mutants showed increased growth of an avirulent strain of *Pst* DC3000 carrying *AvrRpt2* [74, 92]. In the SA-deficient *eds5* mutant, the resistance to *Pst* DC3000 *AvrRpt2* is also impaired [91, 93]. In addition, the *npr1-1 npr4-4D* double mutant is more susceptible to *Pst* DC3000 *AvrRpt2* and *Pst* DC3000 *AvrRps4* [73]. However, the cell death in *eds5-3* and *npr1-1 npr4-4D* is increased following *Pst* DC3000 infection [91], and SA-pretreatment blocks the HR induced by *P. syringae* pv. *maculicola* (*Psm*) ES4326 *AvrRpm1* [94], indicating that SA negatively regulates ETI-induced cell death. ENHANCED DISEASE SUSCEPTIBILITY 1 (EDS1) and PHYTOALEXIN DEFICIENT 4 (PAD4) are two key signaling components that form a complex downstream of Toll/interleukin-1 receptor (TIR)-containing proteins to perceive the pRib-AMP/ADP signal generated by TIR enzymatic activity, and SA treatment induces the expression of both *EDS1* and *PAD4* [95–99]. Moreover, SA treatment upregulates many sensor NLR genes such as *resistance to P. syringae pv. maculicola 1* (*RPM1*), *zygotic arrest 1* (*ZAR1*), *soybean SMV resistance cluster 7* (*SRC7*), *AhRRS5*, *aspergillus flavus-induced NBS-LRR gene 4* (*AhRAF4*), *Dendrobium officinale resistant to P. syringae 2* (*DofRPS2*) and *Zea mays nucleotide-binding site encoding gene 25* (*ZmNBS25*), as well as two helper NLR genes *activated disease resistance 1-like 1* (*ADR1-L1*) and N *requirement gene 1b* (*NRG1b*) [100]. These findings support the crucial role of SA in boosting ETI.

Beyond its role in PTI and ETI, SA is indispensable and sufficient to induce SAR, as *Arabidopsis* and tobacco transgenic plants overexpressing *NahG* are defective in SAR, while exogenous application of SA can induces SAR [74, 76, 101]. However, analysis of chimeric tobacco generated by grafting combinations of wild type and *NahG*-expression rootstocks and scions revealed that although SA accumulation is required for SAR, SA is unlikely the long-distance signal moving from local infection sites to the distal leaves [75]. The mobile signal of SAR had been elusive for decades, and two independent studies revealed that N-hydroxypipecolic acid (NHP) likely acts as the SAR mobile signal in 2018 [102–104]. NHP accumulates both locally and systemically after pathogen infection. The induction of NHP biosynthesis in the local tissue requires activation of SA signaling, as the expression of NHP biosynthesis genes cannot be activated in the *npr1-1 npr4-4D* double mutant [73]. ChIP-qPCR analysis revealed that the NHP biosynthetic genes are not directly targeted by NPR1 and NPR3/NPR4 via TGA2/TGA5/TGA6, but rather by the SA-responsive SARD1 and CBP60g transcription factors [73]. Remarkably, application of the NHP precursor pipecolic acid (Pip) or NHP was unable to induce resistance to *P. syringae* and *Hyaloperonospora arabidopsidis* (*Hpa*) Noco2 in *sid2*, *npr1,* and *npr4-4D* mutants [102], suggesting that SA also acts downstream of NHP in plant immunity [73, 102, 103, 105]. Increasing evidence suggest that SA and NHP form a mutual amplification loop to boost immunity [106].

## Biosynthesis and metabolism of JA

**Figure 2:**
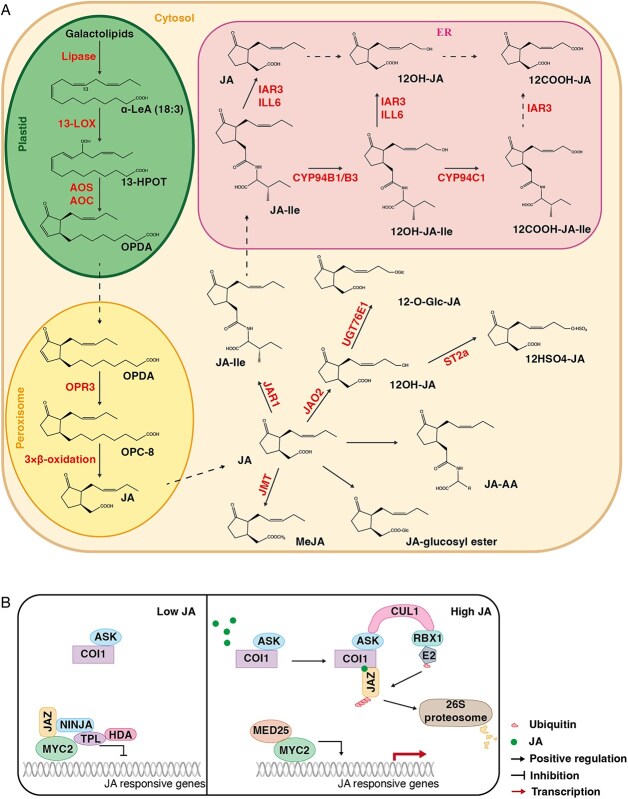
The synthesis, metabolism and signaling of JA. (A) The 12-oxo-phytodienoic acid (OPDA) is formed from α-linolenic acid (α-LeA) generated from galactolipids in plastids. In peroxisomes, OPDA is converted into JA, which is then transported to the cytosol. To turn over and modulate JA levels, JA can be converted to diverse derivatives, including the bioactive JA-Ile, which then enters nucleus to activate JA signaling. (B) In nucleus, the F-box subunit of SCF^COI^ E3 ligase COI perceives the bioactive JA-Ile and recruits JAZ proteins for ubiquitination and degradation. The degradation of transcription repressors, JAZs, results in release of transcription factors and activation of JA signaling. 13-LOX, 13-LIPOXYGENASE; AOS, ALLENE OXIDE SYNTHASE; AOC, ALLENE OXIDE CYCLASE; OPR3, OPDA REDUCTASE3; JAR1, JA-AMINO ACID SYNTHETASE; JMT, JA CARBOXYL METHYLTRANSFERASE; JAO2, JASMONATE-INDUCED OXYGENASES 2; ILL6, IAA-LEUCINE RESISTANT (ILR)-LIKE GENE 6; TAR3, IAA-ALANINE RESISTANT 3 (IAR3); ST2a, 12-OH-JA SULFOTRANSFERASE.

Biosynthesis of JAs has been investigated in a variety of plants [107–109]. In *Arabidopsis*, this pathway initiates in the plastid with the release of α-linolenic acid (18:3, α-LeA) from galactolipids by phospholipases or lipases, such as DEFECTIVE IN ANTHER DEHISCENCE 1 (DAD1), DONGLE (DGL), and PHOSPHOLIPASE A-TYPE 1 γ1 (PLA1γ1) (Fig. 2A) [110–113]. α-LeA is oxygenated by 13-LIPOXYGENASES (13-LOXs) to produce (13S)-hydroperoxyoctadecatrienoic acid (13-HPOT) [114]. Next, 13-HPOT is converted to 12-oxo-phytodienoic acid (OPDA) in two steps, oxidation by the cytochrome P450 enzyme ALLENE OXIDE SYNTHASE (AOS) and subsequent cyclization by the ALLENE OXIDE CYCLASE (AOC) [115–120]. Production of JA from OPDA occurs in the peroxisome, where the cyclopentenone ring of OPDA is reduced by a 12-oxophytodienoate reductase to produce 3-oxo-2-(2-pentenyl)-cyclopentane-1-octanoic acid (OPC-8) [121, 122]. Subsequent removal of six carbons from the carboxyl side chain of OPC-8 via three rounds of β-oxidation gives rise to JA [123]. An OPDA REDUCTASE 3 (OPR3)-independent pathway for JA synthesis has also been reported in the complete loss-of-function *opr3–3* mutant [124, 125]. In this alternative pathway, the OPDA enters the β-oxidation pathway to produce 4,5-ddh-JA, which is subsequently reduced to JA by OPR2 [124, 125].

JA can be converted to a variety of derivatives with increased, reduced or complete loss of bioactivity (Fig. 2A). At least twelve metabolic pathways converting JA or its derivates are known so far. Conjugation of L-Ile to JA by JASMONOYL-ISOLEUCINE SYNTHETASE (JAR1) gives rise to (+)-7-*iso*-JA-L-Ile (hereafter, JA-Ile), which is the most bioactive form of JA. In addition to JA-Ile, four other JA-amino acid conjugates, (+)-7-*iso*-JA-L-Ala, (+)-7-*iso*-JA-L-Val, (+)-7-*iso*-JA-L-Leu, and (+)-7-*iso*-JA-L-Met also function as endogenous JA bioactive molecules with distinct activities promoting the interaction of JAZ and SCF^COI1^ [126, 127]. The active JA-Ile can be hydroxylated by several members of the CYP94 gene family to produce the inactive 12OH-JA-Ile, which is then further oxidized by CYP94C1 to yield 12COOH-JA-Ile [128–130]. Besides, both JA-Ile and 12OH-JA-Ile can be cleaved by IAA-LEUCINE RESISTANT (ILR)-LIKE GENE 6 (ILL6) and IAA-ALANINE RESISTANT 3 (IAR3) to form JA and 12OH-JA, respectively [131, 132]. In addition to CYP450s, the 2-oxoglutarate-dependent dioxygenase (2OGD) enzyme, JASMONATE OXIDASE 2 (JAO2), can hydroxylate JA to 12OH-JA as well [133]. Methylation of JA by JA carboxyl methyltransferase (JMT) yields methyl jasmonate (MeJA), a diffusible intercellular signal transducer [134]. Apart from the compounds mentioned above, a number of other JA derivatives have been identified *in planta*, including JA glucosyl ester, *cis*-jasmone, 12-*O*-glucosyl-JA, 12-HSO_4_-JA, 12-O-glucosyl-JA–Ile, JA–Ile-glucosyl ester, and JA–Ile methyl ester [108, 109].

## JA signaling

The F-box protein CORONATINE INSENSITIVE1 (COI1) acts as the JA receptor to stimulate the expression of JA-responsive genes (Fig. 2B) [135–137]. In JA signaling, JA-Ile binds COI1 to promote 26S proteosome-mediated degradation of JASMONATE ZIM-DOMAIN (JAZ) proteins, which interact with and suppress downstream transcription factors to repress JA responses [138–142]. Removal of JAZ proteins enables activation of defense responses and other JA-dependent processes [143, 144]. In the absence of JA, JAZ recruits the corepressor TOPLESS (TPL) through the NOVEL INTERACTOR OF JAZ (NINJA) adaptor protein [145]. Upon initial binding of JA-Ile or its analog coronatine (COR), the F-box protein COI1 recruits JAZ proteins to form a co-receptor complex, triggering degradation of JAZ proteins by the SCF^COI1^ E3 ligase complex, which consists of SKP1 (ASK1 or ASK2), CULLIN1 (CUL1), RING-BOX PROTEIN 1 (RBX1), and COI1 [140, 141, 146–150]. As a result, the degradation of JAZs releases of the repression of downstream transcription factors, thereby activating of JA signaling [141, 142, 151]. Failure to degrade JAZs leads to constitutive repression of their targets and prevents the expression of JA-dependent genes [139, 140].

The JAZ proteins target numerous transcriptional activators and repressors to regulate various biological processes, including wound responses, defense against insects and microbial pathogens, stamen development and seed production, root hair growth, trichome formation, oxidative stress tolerance, tolerance to freezing and salt, anthocyanin biosynthesis, and crosstalk with other hormones [109]. One of the best-characterized targets of JAZs is the basic helix–loop–helix (bHLH) transcription factor MYC2 (Fig. 2B), which serves as a central transcription factor in JA signaling and regulates a large number of JA-responsive genes [151, 152]. MYC2 regulates the transcription of target genes by forming homodimers or heterodimers with its functionally redundant homologs, MYC3 or MYC4 [153–155]. Interaction between the Jas motif of JAZ proteins and the JAZ interaction domain (JID) of MYC2 restricts access of MYC2 to the mediator subunit MEDIATOR 25 (MED25) and represses expression of downstream genes [146, 156, 157].

## JA in plant immunity

In contrast to SA, which activate defense responses biotrophic and hemibiotrophic pathogens, JAs primarily activate plant defenses against herbivores and necrotrophic pathogens. Exogenous application of JAs has been shown to boost plant defense responses in many plant species. Arabidopsis plants treated with MeJA display reduced susceptibility to *Alternaria brassicicola*, *Botrytis cinerea,* and *Plectosphaerella cucumerina* [158]. Similarly, pretreatment of MeJA reduces disease development by root knot nematode and *B. cinerea* in tomato [159, 160]. In addition, MeJA application increases resistance to *F. graminearum* [161, 162] and *Blumeria graminis* f. sp*. tritici* in susceptible wheat varieties [163]. Furthermore, cucumber treated with JA shows enhanced resistance to the vegetable leafminer *Liriomyza sativae* [164]. In rice, exogenous supply of MeJA also induces defense against *Meloidogyne graminicola* [165]. These studies suggest that the central role of JA in defense responses is conserved in plants [166–168].

Plants rapidly accumulate JA upon insect feeding, pathogen challenges, or mechanical wounding, thereby triggering large-scale transcriptional reprogramming [169]. As a result, plants with impaired JA biosynthesis or perception typically exhibit drastically reduced resistance [170–174]. For instance, the JA-deficient Arabidopsis mutants *aos* and *opr3* are significantly more susceptibility to cabbage loopers and *B. cinerea* [174]. The Arabidopsis acyl-coenzyme A oxidase (ACX) *acx1 acx5* double mutant with severe JA deficiency also showed decreased resistance to *Trichoplusia ni* larvae [175]. In addition, Arabidopsis *jar1* mutants exhibit increased susceptibility to pathogens and insects [135, 176]. Moreover, mutation of BFP1, which promotes the degradation of JAOs, results in significantly lower JA levels and increased susceptibility to *B. cinerea* [177]*.* Disrupting JA signaling also leads to reduced resistance in plants. The *coi1* mutant is deficient in JA-induced response and more susceptible to pests [147, 178], whereas the *jaz* quintuple mutant *jaz1*/*3*/*4*/*9*/*10* exhibits constitutive JA response and enhanced defense [144]. Similar to *coi1*, the *myc2 myc3 myc4* triple mutant is impaired in activation of JA-mediated responses, leading to reduced susceptibility to *P. syringae* and compromised resistance to *Spodoptera littoralis* [153]. Furthermore, failure to remove the H3K27me3 of JA-responsive genes, including *PDF1.2*, increases the susceptibility to *B. cinerea* in *yb2 yb3* [179]*.*

Genetic studies in tomato, rice, and maize further highlight the essential role of JA in defense against herbivore attacks and pathogen infection. In tomato, the JA-deficient mutant *prosystemin-mediated responses2* (*spr2*) is compromised in defense against insect attacks and *B. cinerea*, and more sensitive to the root knot nematodes [180–182]. Another JA-deficient tomato mutant *defenseless-1* (*def1*) also shows enhanced susceptibility to *Fusarium oxysporum*, *Verticillium dahlia,* and *B. cinerea* [183, 184]. In addition, loss of function of JA-Ile receptor in tomato leads to 100% mortality from root rot disease caused by the oomycete pathogen *Pythium* [185]. In rice, blocking JA biosynthesis by disrupting *OsAOC* compromises the resistance to *M. oryzae* [186]. In maize, loss of function of both OPR7 and OPR8 leads to reduced production of JAs, as well as diminished resistance to *Pythium* and insects [187]. Furthermore, disrupting the interaction between LIGULELESS1 (LG1) and ZINC-FINGER PROTEIN EXPRESSED IN INFLORESCENCE MERISTEM (ZIM1) in maize prompts COIa-mediated ZIM1 degradation, which enhances the aphid resistance [188].

In general, the JA response can be subdivided into two branches [6]. The ERF (also known as JA/ET) branch is activated by necrotrophic pathogens and is co-regulated by ethylene (ET) and members of APETALA 2 (AP2)/ERF transcriptions factors, such as OCTADECANOID-RESPONSIVE ARABIDOPSIS AP2/ERF 59 (ORA59), and ETHYLENE RESPONSE FACTOR 1 (ERF1) [189, 190]. The MYC branch, on the other hand, is primarily associated with the wound response and defense against herbivores [6]. Insect feeding activates *MYC2* transcription, which in turn stimulates the expression of the MYC2-branch marker gene *VEGETATIVE STORAGE PROTEIN 2* (*VSP2*) and suppresses the expression of the ERF branch marker gene *PLANT DEFENSIN 1.2* (*PDF1.2*) [6]. In contrast, ERF1 prevents the induction of wound response genes, including *VSP2* [151]. The interplay between MYC2 and ERF determines the appropriate responses to overcome different stresses.

To counteract JA-mediated plant defenses, herbivores have evolved sophisticated approaches to manipulate JA responses to weaken and evade plant defense. For instance, the HARP1 effector secreted from *Helicoverpa armigera* binds JAZ proteins and blocks signal transduction by preventing COI1-mediated JAZ degradation, thereby reducing the insect resistance and JA-dependent wounding responses [191]. Similarly, HIGHLY ACCUMULATED SECRETORY PROTEIN 1 (HAS1) secreted by *H. armigera* also interferes host defense by interacting with MYC3 and MYC4 [192]. In addition, the antibiotic biosynthetic monooxygenase (Abm) of *M. oryzae* converts free JA into 12OH-JA, which is released to inhibit JA activity and compromise the host immune response [193].

Similar to SA signaling in SAR, JA signaling is also essential for induced systemic resistance (ISR), which is activated by beneficial microbes such as *Pseudomonas* spp., *Bacillus* spp., *Streptomyces* spp., and *Trichoderma* spp. [194]. ISR renders uninfected plant tissues resistant to a broad-spectrum attackers including biotrophic and necrotrophic pathogens, as well as herbivores [194]. In mutants such as *jin1*, *jar1,* and *coi1*, this resistance is severely compromised [195, 196]. Data from grafting assays using a JA-deficient mutant and a JA response mutant suggest that JA, or its related compounds produced from the damaged local leaves, may act as the ISR mobile signal [197]. JA transporters (JAT), AtJAT3 and AtJAT4, have been shown to participate in the long-distance translocation of JA from one leaf to another in wound-induced systemic resistance, further emphasizing the critical role of JA in ISR [198]. In addition to ISR, JA signaling is also required for plants to develop enhanced immunity in mycorrhiza-induced resistance (MIR) response that is triggered by arbuscular mycorrhizal fungi (AMF) [199, 200].

## The crosstalk between SA and JA in plant defense responses

Both SA and JA involve a network of defense-related genes encoding transcription factors, biosynthetic enzymes and receptors etc., which are interconnected to coordinate defense against pathogen invasion and developmental signals. The SA-mediated defense responses play crucial roles in both local and systemic resistance against biotrophs [10], while resistance conferred by necrotrophic pathogens requires JA signaling [185, 201]. To fend off pathogens with different virulence strategies, plants have evolved complex defense mechanism, where crosstalk between these signaling pathways optimizes the response to individual attackers.

**Figure 3:**
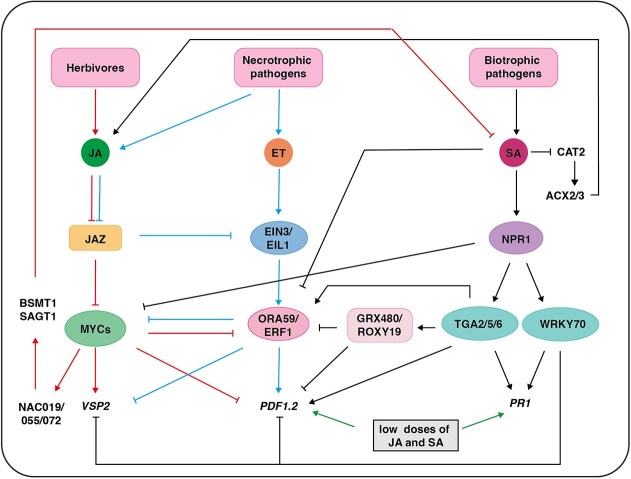
Simple schematic of the crosstalk between SA and JA. Arrows, positive regulation; inhibition lines, negative regulation; black lines, SA signaling pathway; red lines, MYC branch of JA signaling pathway; blue lines, ERF branch of JA signaling pathway; green lines, synergistic interactions between SA and JA.

The interaction between SA and JA is mutually antagonistic in most cases [202–204]. After the initial observation that pretreatment with the SA-related compound aspirin prevents wound-induced JA accumulation in tomato, SA was found to suppress JA biosynthesis in many plants [202, 204]. In Arabidopsis *NahG* transgenic plants, which cannot accumulate SA, both JA levels and the expression of JA-responsive genes were elevated when infected by *Pst* DC3000 [205]. They also accumulate higher levels of JA than the wild type after herbivore feeding [206]. Similarly, JA levels in the *sid2-2* mutant are much higher than in the wild type as SA-mediated repression of ACX2 and ACX3 is abolished [207]. CATALASE2 (CAT2) stimulates the activity of ACX2 and ACX3 to increase JA accumulation, and SA inhibits CAT2 activity to reduce JA production (Fig. 3) [207]. Besides endogenous SA, exogenous application of SA and its analogs have similar effects. Treatment with benzothiadiazole (BTH), a synthetic SA analog, resulted in increased weight gain of *Spodoptera frugiperda* (J.E. Smith) (FAW). In contrast, application of JA led to reduced growth of FAW on cotton and soybean [208]. Interestingly, infection with hemibiotrophic *P. syringae* rendered plants more susceptible to the necrotrophic *A. brassicicola* adjacent to the site of initial infection [209]. However, in systemic tissues, *A. brassicicola* infection was not affected, suggesting that the tradeoff between SA and JA signaling may be spatially controlled [209]. Moreover, antagonism between the SA and JA response pathways was even shown to remain active in the next generation. For example, the progeny of *Pst* DC3000-inoculated Arabidopsis displayed reduced responsiveness of JA-inducible genes and enhanced susceptibility to necrotrophic pathogens [210].

Increasing evidence indicates that activation of SA signaling inhibits the MYC branches of JA pathways. The SA receptor NPR1 has been reported to physically interact with MYC2 and its homologs to prevent the activation of JA signaling (Fig. 3) [211]. In Arabidopsis *npr1-1* mutants, enhanced JA-responsive gene expression and increased JA levels were observed upon *Pst* DC3000 infection [205]. In the presence of both SA and JA, NPR1 is recruited to the JA-responsive promoter regions to suppress gene transcription by disrupting the interaction between MYC2 and MED25 [211]. Interestingly, the enhanced susceptibility to *Psm* ES4326 in *npr1-3* is recovered by the *myc2 myc3 myc4* triple mutant, genetically supporting that NPR1 alleviates COR-induced pathogenicity by negatively regulating the activity of MYC activators [211]. In agreement, knockout of tomato *NPR1* leads to activation of JA signaling and increased resistance to *B. cinerea* [212].

SA also inhibits the ERF branch of JA pathway in an NPR1-independent manner when applied together with ET, suggesting that other regulators are involved in the control the ERF branch [213]. Notably, the induction of *PDF1.2* by a combination of JA and the ET precursor 1-aminocyclopropane-1-carboxylic acid (ACC) is blocked in the *tga256* triple mutant and enhanced in transgenic lines overexpressing *TGA5* [214]. However, the TGACG motif is not necessary for *PDF1.2* promoter activity in the JA-ACC-treated plants [214]. Further analysis showed that the SA-responsive glutaredoxin GRX480/ROXY19 functions as a TGA2-interacting transcriptional repressor to inhibit the expression of *PDF1.2* (Fig. 3) [215–217], but TGA2, TGA5, and TGA6 are also required for the induction of *ORA59* by ACC (Fig. 3) [217]. ChIP assays showed that TGA factors directly bind to the TGACGT motif on the *ORA59* promoter [217]. Since ORA59 is a master regulator that controls the expression of downstream genes in the ERF branch, it was proposed that TGA factors bind to the ORA59 promoter to induce its transcription, which in turn activates *PDF1.2* expression [214–217]*.* In the presence of elevated SA, SA-induced GRX480/ROXY19 is recruited to the TGA2/5/6 binding site in the *ORA59* promoter, where it represses transcription, thereby reducing the *PDF1.2* expression [214–217].

Several other SA-induced transcriptional regulators, such as WRKY70, have been shown to negatively affect JA-responses. WRKY70 serves as a critical regulatory node for SA-JA crosstalk [218]. Expression of *WRKY70* is strongly induced by SA and repressed by JA [218]. Overexpression of *WRKY70* results in constitutive expression of *PR* genes and increased resistance to biotrophic pathogens but simultaneously causes attenuated expression of JA-inducible genes and compromised resistance to necrotrophic *A. brassicicola* (Fig. 3) [218, 219]. In contrast, down-regulation or knockout of *WRKY70* activates JA-inducible genes expression and promotes resistance to this pathogen [218, 219]. Interestingly, JA levels are not altered in either gain- or loss-of-function mutants of *WRKY70* [218, 219]. Moreover, WRKY70 has also been shown to repress *SARD1* expression in the absence of pathogens [220], which is consistent with the elevated SA levels observed in the *wrky70* and *wrky70 wrky54* mutants without infection [221]. These results indicate that WRKY70 has a pivotal role in determining the balance between SA- and JA-dependent plant immunity.

Not only SA inhibits JA responses but also JA suppresses SA-mediated immunity. In Arabidopsis *coi1* and *jin1*/*myc2* mutants, there is a significantly greater increase in SA and *PR-1* expression levels after *Pst* DC3000 infection [222, 223]. Robust resistance to *P*. *syringae* was observed in these JA-insensitive mutants [147, 222, 223]. Mutation of *MpCOI1* confers resistance to *F. oxysporum* in the liverwort *Marchantia polymorpha* [203], whereas ectopic overexpression of *AtCOI1* in the *mpcoi1* mutant increases the susceptibility to *Pst* DC3000, indicating that the JA-mediated repression of SA signaling may be conserved in land plants [224].

Consequently, some pathogens have evolved strategies to manipulate the antagonistic interactions between SA and JA, which suppress SA-mediated immunity to promote virulence. The most well-known example of a pathogen manipulating JA pathways is the action of coronatine (COR), produced by *P. syringae*, which mimics JA-Ile to trigger SCF^COI1^-mediated JAZ degradation, resulting in the release of MYC2 [225]. MYC2 then positively regulates the expression of three closely related NAC transcription factors, ANAC019, ANAC055, and ANAC072, which play critical roles in regulating SA biosynthesis and metabolism [149]. In the *anac019/055/072* triple mutant, the susceptibility to *Psm* ES4326 triggered by COR is significantly reduced [149]. Through activation of two SA metabolic enzyme genes, *BSMT1* and *SA GLUCOSYL TRANSFERASE GENE 1* (*SAGT1*), ANAC019/055/072 promote the conversion of SA into its inactive forms, thereby blocking SA signaling [149]. Although these NACs have been reported to possess transactivation activity [226], the *ICS1* expression is elevated in the *nac* triple mutant [149]. This suggests that they may also function as transcriptional repressors by interacting with or recruiting different transcription factors to suppress SA biosynthesis [149]. In tomato, a homolog of these NACs, *JASMONIC ACID2 LIKE* (*JA2L*), was also shown to dampen SA accumulation by promoting the conversion of SA into MeSA in a COR-dependent manner [227].

Notably, some herbivores can also exploit the SA-JA antagonism to suppress JA-mediated defense. The saliva of Colorado potato beetle contains numerous bacteria, which can trigger SA-mediated immunity and inhibit JA-mediated defense through the SA-JA antagonism [228]. Similarly, the mealybug *Phenacoccus solenopsis* also employs symbiotic microbes in its saliva to activate SA signaling while simultaneously repressing JA-regulated defenses [229]. In addition, the glucose oxidase in the saliva of *Spodoptera exigua* can elicit an SA burst in *Nicotiana attenuata*, thereby antagonizing the JA burst [230].

It is worth noting that in the intricate network of plant defense responses, SA and JA exhibit not only antagonistic interactions but also synergistic effects. A transient synergistic enhancement in the expression of JA-associated genes *PDF1.2* and *Thi1.2* in Arabidopsis and SA-associated gene *PR1a* in tobacco was observed when JA and SA were applied simultaneously at low concentrations (Fig. 3) [231]. In addition, many genes are commonly induced by treatment with either SA or JA in Arabidopsis [232]. SA and JA signaling are sometimes simultaneously activated during ETI and PTI. In the *dde2*/*ein2*/*pad4*/*sid2*-quadruple mutant, which is deficient in SA, JA, and ET signaling, the level of immunity triggered by flg22 (PTI) against *Pst* DC3000 was diminished to 20% of that in the wild type [233]. Moreover, ETI-triggered by AvrRpt2 was also reduced to 20% and 50% in this mutant, respectively [233]. Spatiotemporal analysis indicates that SA and JA signaling are concurrently activated in distinct concentric domains in RPS2-triggered immunity [90]. SA-mediated defense responses are activated in the SA zone, while the JA-active domain outside the SA infection foci protects living cells around the HR cell death area [90].

Notably, SA-JA cooperation is also observed in other species. In poplar, both SA and JA accumulate to greater amounts in leaves after infection with the biotrophic rust fungus *Melampsora larici-populina* and herbivores [234]. Transgenic black poplar with hyperaccumulated SA displayed elevated JA content, and treatment with either MeSA or MeJA increased the levels of both endogenous JA and SA [235]. Synergism between SA and JA was also reported in rice. For example, mutation of *Pi21* or *ERF922* led to enhanced SA and JA defense responses, resulting in increased resistance to rice blast and bacterial blight [8]. Additionally, the rice EIN3/EIL homolog, OsEIL3, activates SA and JA biosynthesis and signaling, enhancing resistance following infection by hemibiotrophic and biotrophic pathogens [9]. However, infections with necrotrophic pathogens repress SA and JA biosynthesis and signaling, compromising plant resistance [9]. These findings suggest that the synergistic SA-JA interactions may provide a stronger and more efficient defense against pathogens with different life styles [9]. In addition, two other independent studies showed that a large number of SA-induced genes are also upregulated by JA [236, 237]. However, to what extent the SA-JA crosstalk is conserved in different plants still requires further investigation.

## Future perspectives

Plant immunity is regulated by a sophisticated network of cross-communicating phytohormones, where SA and JA play dominant roles. Although tremendous progress has been made in understanding their biosynthesis, metabolism, physiology, perception and downstream signaling, and interactions with each other, many questions remain. For example, how SA is synthesized via the PAL pathway, and how SA regulates the transcriptional repression activities of NPR3/NPR4 and transcriptional activation activity of NPR1, are still unclear. Furthermore, the mechanism by which NHP activates SA biosynthesis and signaling; how SA coordinates with JA in ETI, PTI, and ISR, how spatiotemporal dynamics of SA-JA crosstalk are regulated; and whether the SA-JA crosstalk can be disconnected still need to be addressed.
